# Identification of a *cis*-acting DNA–protein interaction implicated in singular *var* gene choice in *Plasmodium falciparum*

**DOI:** 10.1111/cmi.12004

**Published:** 2012-09-04

**Authors:** Nicolas M B Brancucci, Kathrin Witmer, Christoph D Schmid, Christian Flueck, Till S Voss

**Affiliations:** 1 Department of Medical Parasitology and Infection Biology, Swiss Tropical and Public Health Institute4051, Basel, Switzerland; 2 University of Basel4003, Basel, Switzerland

## Abstract

*Plasmodium falciparum* is responsible for the most severe form of malaria in humans. Antigenic variation of *P. falciparum* erythrocyte membrane protein 1 leads to immune evasion and occurs through switches in mutually exclusive *var* gene transcription. The recent progress in *Plasmodium* epigenetics notwithstanding, the mechanisms by which singularity of *var* activation is achieved are unknown. Here, we employed a functional approach to dissect the role of *var* gene upstream regions in mutually exclusive activation. Besides identifying sequence elements involved in activation and initiation of transcription, we mapped a region downstream of the transcriptional start site that is required to maintain singular *var* gene choice. Activation of promoters lacking this sequence occurs no longer in competition with endogenous *var* genes. Within this region we pinpointed a sequence-specific DNA–protein interaction involving a *cis*-acting sequence motif that is conserved in the majority of *var* loci. These results suggest an important role for this interaction in mutually exclusive locus recognition. Our findings are furthermore consistent with a novel mechanism for the control of singular gene choice in eukaryotes. In addition to their importance in *P. falciparum* antigenic variation, our results may also help to explain similar processes in other systems.

## Introduction

Many unicellular pathogens use antigenic variation to escape adaptive immune responses in the host. The widespread occurrence of this strategy in evolutionary distant species underscores its key role in pathogen survival and spreading. While the underlying control pathways are highly diverse in different systems, both mechanistically and in terms of complexity, antigenic variation is defined by two basic concepts. First, the antigens are encoded by gene families, the members of which are expressed in a mutually exclusive manner. Second, switches in the expression of individual members lead to antigenic variation of surface-exposed antigens. In several medically important pathogens such as *Borrelia* spp., *Neisseria* spp., *Giardia lamblia*, *Plasmodium falciparum* and *Trypanosoma brucei*, this paradigm of clonal phenotypic variation reaches a remarkable yet poorly understood level of sophistication (Deitsch *et al*., 2009; Dzikowski and Deitsch, 2009; Morrison *et al*., 2009; Prucca and Lujan, 2009).

The apicomplexan parasite *P. falciparum* causes several hundred million malaria cases and close to one million deaths annually (World Health Organization, 2010). Malaria-associated morbidity and mortality is a result of the intra-erythrocytic developmental cycle (IDC) where repeated rounds of parasite invasion into red blood cells (RBCs) are followed by intracellular maturation and replication. During this stage of infection parasites expose the major virulence factor *P. falciparum* erythrocyte membrane protein 1 (PfEMP1) on the RBC surface (Leech *et al*., 1984). This highly polymorphic antigen, encoded by the 60-member *var* gene family, undergoes antigenic variation to facilitate chronic infection and transmission (Biggs *et al*., 1991; Roberts *et al*., 1992; Smith *et al*., 1995; Su *et al*., 1995; Gardner *et al*., 2002). Furthermore, PfEMP1 mediates sequestration of infected RBC aggregates in the microvasculature of various organs and is thus directly responsible for severe outcomes, including cerebral and placental malaria (MacPherson *et al*., 1985; Pongponratn *et al*., 1991; Baruch *et al*., 1996; Gardner *et al*., 1996; Reeder *et al*., 1999; Beeson and Duffy, 2005).

*var* genes are transcribed by RNA polymerase II (RNA polII) in ring-stage parasites during the first half of the IDC (Scherf *et al*., 1998; Dzikowski *et al*., 2006; Kyes *et al*., 2007). Notably, only one *var* gene is transcribed at any time while all other members are silenced (Scherf *et al*., 1998). Switches in *var* gene transcription, and consequently antigenic variation of PfEMP1, are independent of detectable recombination events and occur by *in situ var* gene activation (Scherf *et al*., 1998). *var* gene silencing is explained by the fact that all *var* genes are positioned in subtelomeric and some chromosome-internal heterochromatic regions (Gardner *et al*., 2002; Flueck *et al*., 2009; Lopez-Rubio *et al*., 2009; Salcedo-Amaya *et al*., 2009). These chromosomal domains are uniformly enriched in histone 3 lysine 9 tri-methylation (H3K9me3) and *P. falciparum* heterochromatin protein 1 (PfHP1) (Flueck *et al*., 2009; Lopez-Rubio *et al*., 2009; Perez-Toledo *et al*., 2009; Salcedo-Amaya *et al*., 2009). The presence of these epigenetic marks is directly linked to *var* gene silencing (Chookajorn *et al*., 2007; Lopez-Rubio *et al*., 2007; Perez-Toledo *et al*., 2009). In contrast, the active *var* locus is associated with H3K9ac and H3K4me2/me3 instead (Lopez-Rubio *et al*., 2007). Interestingly, singular *var* gene activation is linked to locus repositioning into a dedicated perinuclear expression site (Duraisingh *et al*., 2005; Ralph *et al*., 2005; Marty *et al*., 2006; Voss *et al*., 2006; Dzikowski *et al*., 2007). While the mechanisms underlying this process are largely unknown, a recent study identified a critical role for nuclear actin in locus repositioning and mutually exclusive expression (Zhang *et al*., 2011). Moreover, Volz *et al*. identified a H3K4-specific methyltransferase (PfSET10) and demonstrated its exclusive localization to the active *var* locus suggesting a role for this enzyme in the transmission of epigenetic memory (Volz *et al*., 2012).

In recent years, *var* gene promoters emerged as key components in all layers of *var* gene regulation. Experiments where *var* gene promoters drive transcription of drug-selectable reporter genes have been particularly informative in studying *var* promoter function. In absence of drug selection *var* promoters are predominantly silenced, whereas drug challenge selects for parasites carrying active promoters (Voss *et al*., 2006; 2007). Importantly, this forced activation is sufficient to infiltrate a drug-selectable reporter into the mutual exclusion programme (Voss *et al*., 2006; 2007; Dzikowski *et al*., 2007). In addition to *var* promoters, the *var* intron acts as a cooperative partner in silencing and mutual exclusion (Deitsch *et al*., 2001; Calderwood *et al*., 2003; Gannoun-Zaki *et al*., 2005; Frank *et al*., 2006; Voss *et al*., 2006; Dzikowski *et al*., 2007).

We postulated that transcriptional control of *var* genes may be mediated by unknown sequence information contained within the promoter region. In this study, we developed a functional promoter mapping approach tailored to identify and characterize *var* gene-specific regulatory information. We mapped an autonomous upstream activating sequence (UAS) that is essential for *var* promoter activation. Notably, we also identified a region downstream of the transcriptional start site (TSS) and demonstrate an important role for this element in mutually exclusive promoter recognition. In absence of this sequence *var* promoters are fully active but, unlike wild-type promoters, do not compete with endogenous *var* gene transcription. Within this region we identified a 47 bp motif that interacts in a sequence-specific manner with an unknown nuclear protein. Together, our results show for the first time that the complex regulation of mutually exclusive *var* gene transcription involves functional *cis*-acting modules with intrinsic and position-dependent activities. They are furthermore consistent with a novel mechanism in sustaining singular gene choice in eukaryotes.

## Results

### Functional *var* promoter mapping by bi-directional deletion analysis

**Fig. 1:**
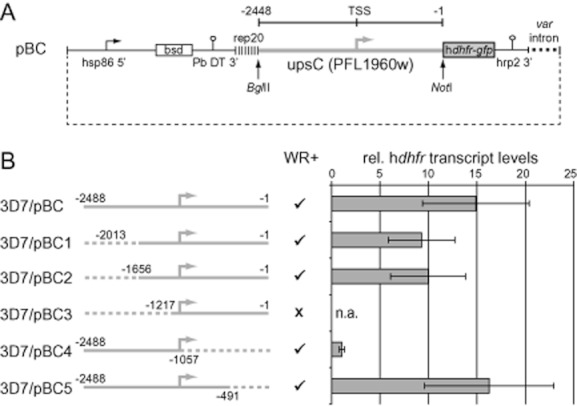
Functional *var* promoter mapping by serial deletion analysis. A. Schematic map of pBC. The PFL1960w upsC upstream sequence controls transcription of h*dhfr-gfp*. The approximate position of the TSS is indicated (Deitsch *et al*., 1999). The *bsd* resistance cassette selects for stably transfected parasites. The *var* intron is indicated by a bold dashed line. pBC descendants were obtained by replacing the upsC promoter with truncated sequences using BglII and NotI. hsp86 5′, *hsp86* promoter; Pb DT 3′, *Plasmodium berghei* *dhfr*-thymidylate synthase terminator; rep20, 0.5 kb TARE6 repeat element; hrp2 3′; histidine-rich protein 2 terminator. B. Activities of full-length and truncated promoters in WR-selected parasites. Deletions are represented by dashed lines. Numbers represent nucleotide positions in relation to the ATG. Successful WR selection is indicated by check marks. Values represent relative h*dhfr-gfp* transcripts normalized against transcription of PF13_0170 (glutaminyl-tRNA synthetase, putative) and plasmid copy number. Values represent the average of three independent experiments (two replicates for 3D7/pBC1 and 3D7/pBC2) (mean ± SEM). n.a., not applicable.

To identify regulatory *var* promoter elements we employed a system suitable to analyse promoter activity in stably transfected parasites. All reporter constructs are based on the parental plasmid pBC (Fig. 1A) where the blasticidin deaminase (*bsd*) resistance cassette selects for stable episomes. A 2.5 kb *var* upsC upstream sequence (PFL1960w) controls transcription of the dual reporter encoding human dihydrofolate reductase fused to green fluorescent protein (h*dhfr-gfp*). A *var* gene intron element is located downstream of the h*dhfr-gfp* cassette to account for its role in *var* gene regulation. A telomere-associated repeat element 6 sequence (TARE6/rep20) is included for improved plasmid segregation (O'Donnell *et al*., 2002). In such a context, homogenous populations carrying active upsC promoters are obtained via selection with the antifolate drug WR99210 (WR) (Voss *et al*., 2006; 2007).

To identify elements involved in promoter activation and mutual exclusion we sequentially truncated the upsC upstream sequence from either the 5′ or 3′ end (Fig. 1B). We chose this bi-directional approach to identify possible functional regions both up- and downstream of the putative TSS. Based on a multiple upsC sequence alignment and the previous experimental mapping of an upsC TSS we expected the TSS of PFL1960w at position −1167 (Deitsch *et al*., 1999; Voss *et al*., 2000). Transfected parasites were challenged with WR and resistant populations were obtained for all but one cell line, 3D7/pBC3 (Fig. 1B). Several attempts to select for WR-resistant 3D7/pBC3 parasites failed showing that the region between −1656 to −1217 comprises an important UAS and/or the core promoter. To test if any of the deletions affected promoter strength we determined relative h*dhfr-gfp* transcript levels in ring-stage parasites by quantitative reverse transcriptase PCR (qRT-PCR). As shown in Fig. 1B, transcript levels in 3D7/pBC1 and 3D7/pBC2 were similar to those in 3D7/pBC indicating that the sequence upstream of −1656 does not contribute to *var* promoter activity. The promoter in pBC5, lacking 491 bp of the 5′ UTR, was also fully active. In contrast, the truncation encompassing bps −1057 to −1 in pBC4 caused a significant reduction in steady-state transcript levels. Hence, this approach identified two regulatory regions, located upstream and downstream of the putative TSS, respectively, which fulfil important roles in *var* promoter function.

### Functional identification of an autonomous upsC upstream activating sequence

**Fig. 2:**
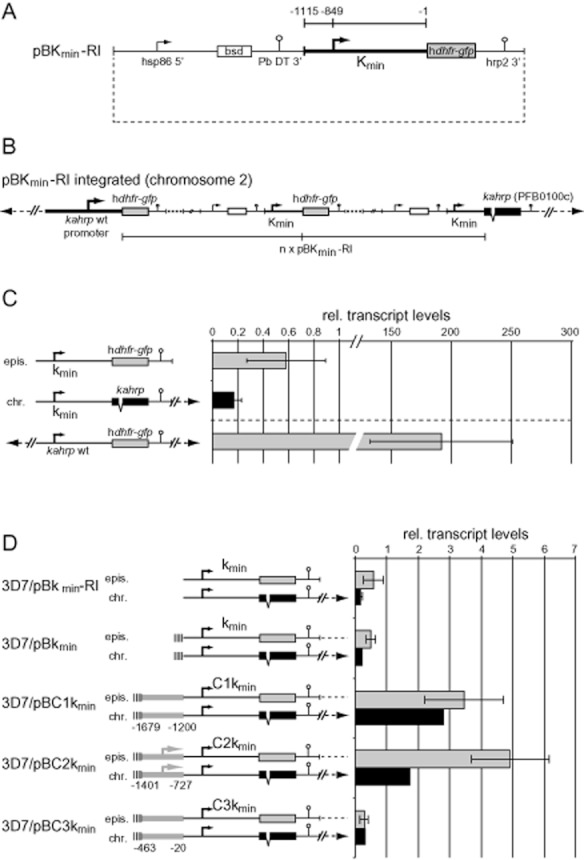
An upsC UAS element activates the minimal promoter K_min__._ A. Schematic map of pBK_min_-RI. The position of the *kahrp* TSS is indicated (Lanzer *et al*., 1992). B. Schematic map of pBK_min_-RI concatamers integrated into the endogenous *kahrp* locus (PFB0100c). C. Comparison of relative transcript levels produced by the episomal (h*dhfr-gfp* transcripts; grey bar) and chromosomal (*kahrp* transcripts; black bar) K_min_ promoters, and the *kahrp* wild-type promoter (h*dhfr-gfp* transcripts; grey bar) in 3D7/pBK_min_-RI parasites. Values are derived from three independent experiments and represent *msp8*-normalized transcripts (mean ± SEM). Values for the episomal K_min_ promoter were additionally adjusted for plasmid copy number. D. Analysis of upsC-K_min_ hybrid promoters. upsC insertions are depicted by bold grey lines. The rep20 element is indicated by a vertical array and the *var* intron by a dashed line. The graph compares relative transcript levels (*msp8*-normalized) produced by the episomal (h*dhfr-gfp* transcripts, grey bars) and chromosomal (*kahrp* transcripts, black bars) K_min_ and upsC-K_min_ hybrid promoters. Values for episomal promoters are derived from three independent experiments (mean ± SEM) and were additionally adjusted for plasmid copy number. Data for 3D7/pBK_min_-RI are identical to those in Fig. 2C.

To learn more about the nature of the putative UAS we set out to analyse its function in the context of a minimal heterologous promoter. We decided to use the knob-associated histidine rich protein (*kahrp*) gene promoter for three reasons. First, the TSS of this gene has been mapped to 849 bp upstream of the ATG (Lanzer *et al*., 1992). Second, similar to *var* genes the timing of *kahrp* transcription peaks in ring-stage parasites. Lastly, the *kahrp* locus is not enriched in H3K9me3/PfHP1 (Flueck *et al*., 2009; Lopez-Rubio *et al*., 2009; Salcedo-Amaya *et al*., 2009), which is an important consideration in order to avoid heterochromatin-mediated masking of autonomous *cis*-acting activities. Hence, we generated plasmid pBK_min_-RI where bps −1115 to −1 of the *kahrp* upstream sequence control transcription of the h*dhfr-gfp* reporter (Fig. 2A). Parasites carrying pBK_min_-RI episomes were readily obtained after transfection. Notably, the disposition of this plasmid to integrate into the endogenous *kahrp* locus allowed us to measure K_min_ activity also in a chromosomal environment. This integration event essentially causes a promoter swap where K_min_ drives expression of the endogenous *kahrp* gene and the endogenous *kahrp* promoter controls transcription of the h*dhfr-gfp* reporter (Figs 2B and S1). Compared with the endogenous full-length *kahrp* promoter, the episomal and chromosomal minimal promoters displayed a 300-fold and 1000-fold reduced activity respectively (Fig. 2C). Hence, K_min_ clearly fulfilled the requirements for a minimal promoter.

We cloned two overlapping fragments containing the putative upsC UAS upstream of K_min_ to create upsC-K_min_ hybrid promoters (pBC1K_min_ and pBC2K_min_) (Figs 2D and S1). The region downstream of the upsC TSS encompassing bps −463 to −20, which has no effect on upsC promoter activity (Fig. 1B), was used as negative control (pBC3K_min_). qRT-PCR analysis revealed that upsC fragments C1 (−1679 to −1200) and C2 (−1401 to −727) consistently activated K_min_ to a similar extent in both the episomal and chromosomal context whereas fragment C3 had no effect. Furthermore, neither the *var* intron nor the rep20 element altered K_min_ activity. Together, these findings corroborate the results obtained with the upsC deletion constructs and are consistent with the presence of a *var* UAS located between bps −1401 and −1217. The fact that this element activates transcription from a heterologous minimal promoter suggests an autonomous, context-independent function in activating RNA polII-mediated transcription.

### Transcriptional initiation from an alternative TSS compensates for the loss of core promoter function

**Fig. 3:**
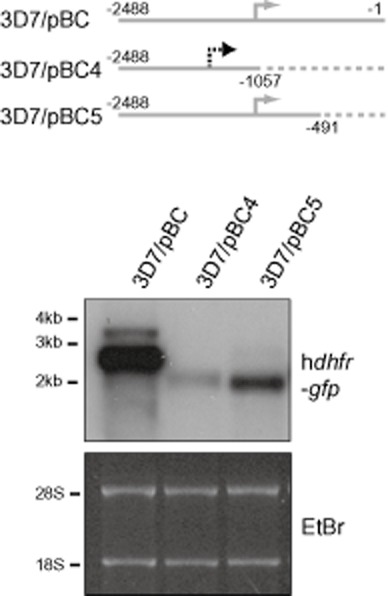
Transcriptional initiation from an alternative upsC upstream TSS. Identification of an alternative upsC upstream TSS (dashed arrow). Full-length and truncated promoters are schematically depicted on top. h*dhfr-gfp* transcript size and abundance was estimated by Northern analysis of total RNA isolated from WR-selected ring-stage parasites. Ethidium bromide-stained 18S and 28S rRNAs serve as loading control.

Here, we investigated the functional region downstream of the putative TSS that is defined by plasmids pBC4 and pBC5 (−1057 to −491). Deletion of this region caused a substantial reduction in steady-state transcripts (Fig. 1B), suggesting it may contain important activating sequences. Northern blot analysis confirmed the reduced abundance of steady-state transcripts in 3D7/pBC4 compared with 3D7/pBC and 3D7/pBC5 (Fig. 3). An independent time-course experiment confirmed these results and excluded the possibility of altered transcriptional timing and/or transcript accumulation in 3D7/pBC4 parasites (Fig. S2). However, these experiments also revealed that the size difference between pBC- and pBC4-derived transcripts was much smaller than expected. In spite of the 1057 bp deletion in the 5′ UTR, pBC4-derived transcripts were larger than those originating from pBC5 where only 491 bp of the 5′ UTR were deleted (Fig. 3). This shows that transcription from the truncated pBC4 upstream sequence initiated from an alternative upstream TSS. Consequently, the reduced steady-state transcript levels observed in 3D7/pBC4 were not related to the deletion of important activating sequences but rather to the loss of proper core promoter function and transcriptional initiation from the natural TSS.

### A regulatory region downstream of the TSS is involved in mutually exclusive *var* gene expression

**Fig. 4:**
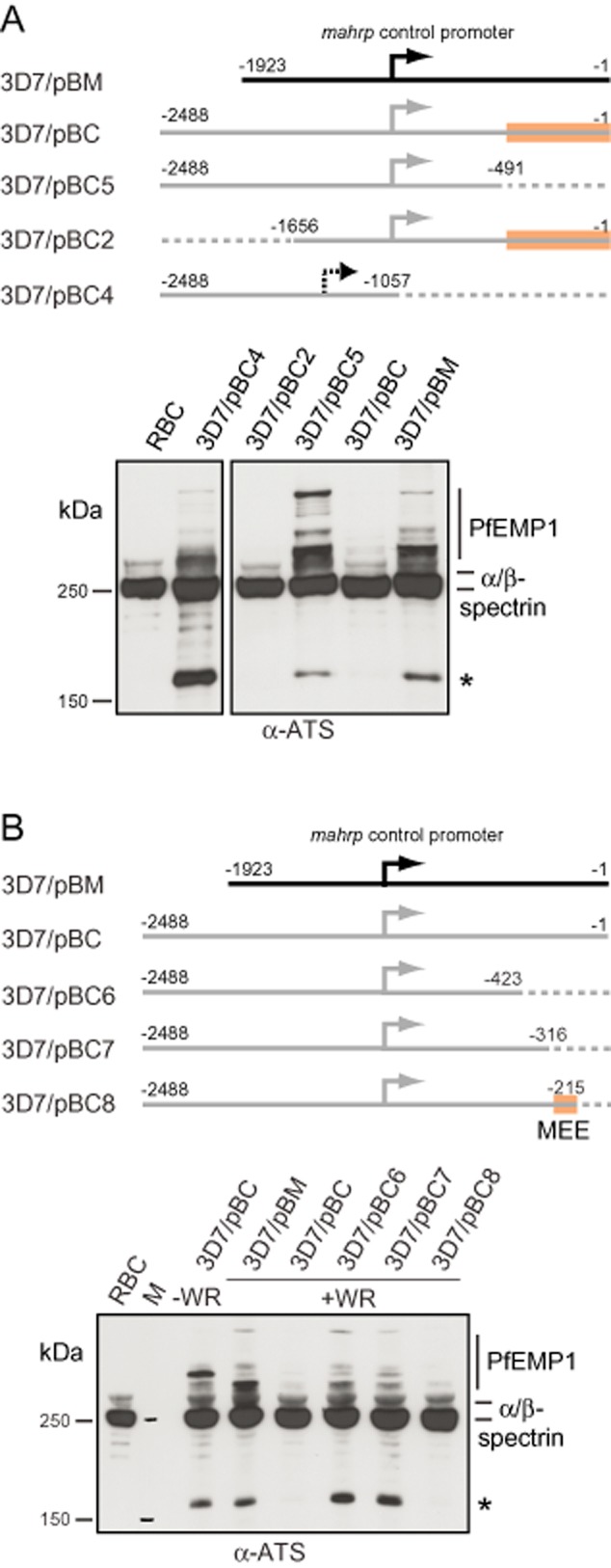
Mutually exclusive activation is mediated by a 101 bp element downstream of the TSS. A. Functional identification of a mutual exclusion element downstream of the TSS. Promoters are schematically depicted on top. pBM is a negative control construct where the *mahrp1* promoter controls h*dhfr-gfp* transcription. upsC sequences are shown in grey. Deletions are represented by dashed lines. The orange box highlights the region required for mutually exclusive activation. PfEMP1 expression in WR-selected trophozoites was monitored by Western blot using antibodies against the conserved ATS domain of PfEMP1 (Duffy *et al*., 2002). The antibody cross-reacts with human spectrin. PfEMP1 is detected at various sizes above 250 kDa. The signal at 160 kDa probably represents smaller PfEMP1 species (asterisk). RBC, uninfected RBCs. B. The mutual exclusion element maps to a 101 bp region downstream of the TSS. The orange box identifies the mutual exclusion element (MEE) located at position −316 to −215. WR-selected 3D7/pBM where the *mahrp* promoter controls h*dhfr-gfp* transcription and WR-unselected 3D7/pBC carrying a silenced upsC promoter served as negative controls. RBC, uninfected RBCs. M, size standard; −WR, unselected; +WR, WR-selected.

Transgenic parasites carrying activated full-length *var* promoters do not transcribe endogenous *var* genes and fail to express PfEMP1 (Dzikowski *et al*., 2006; 2007; Voss *et al*., 2006; 2007; Chookajorn *et al*., 2007; Howitt *et al*., 2009; Witmer *et al*., 2012). This implies that mutually exclusive locus recognition may be mediated by *cis*-acting regulatory sequence elements located in *var* gene upstream regions. To test this hypothesis and to identify such functional elements we investigated if any of the activated truncated promoters escaped mutually exclusive activation. The negative control line 3D7/pBM, in which the unrelated ring stage-specific *mahrp1* promoter controls h*dhfr-gfp* transcription, expressed PfEMP1 at normal levels, whereas parasites of the positive control line 3D7/pBC exhibited the expected PfEMP1 knock-down phenotype (Fig. 4A). PfEMP1 expression was also abolished in 3D7/pBC2 showing that the region ranging from −2488 to −1656 bps upstream of the start codon is not important for mutually exclusive locus recognition. In contrast, 3D7/pBC4 and 3D7/pBC5 parasites expressed PfEMP1 at levels similar to the 3D7/pBM-negative control line. Interestingly, both truncated promoters lack the same 491 bp sequence downstream of the TSS suggesting that this region carries sequence information important for mutually exclusive locus recognition.

To map this region more precisely we cloned three additional truncated upsC sequences in pBC6, pBC7 and pBC8 (Fig. 4B). Similar to the full-length promoter in 3D7/pBC, 3D7/pBC8 parasites failed to express PfEMP1 demonstrating that the pBC8 promoter was activated in a mutually exclusive manner. In contrast, 3D7/pBC6 and 3D7/pBC7 expressed PfEMP1 at levels similar to two negative controls (WR-selected 3D7/pBM and unselected 3D7/pBC) showing that these truncated promoters were not subject to mutually exclusive recognition as already observed for 3D7/pBC5. Together, this series of experiments pinpointed a putative 101 bp mutual exclusion element (MEE) (bps −316 to −215) that drives the upsC promoter into mutually exclusive activation; in absence of the MEE promoters escape this restriction and are activated in parallel to endogenous *var* transcription.

### The mutual exclusion element interacts specifically with an unknown nuclear factor

**Fig. 5:**
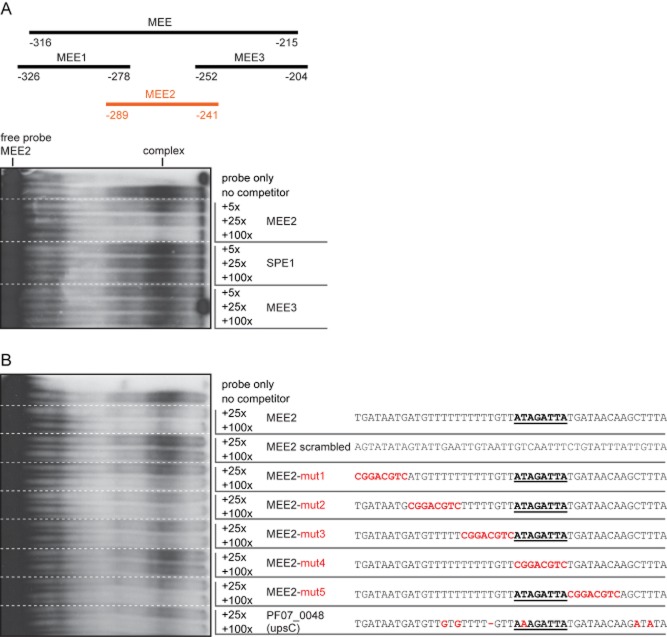
Identification of a sequence-specific DNA–protein interaction implicated in mutual exclusion. A. Identification of a sequence-specific DNA–protein interaction between the 47 bp MEE2 element and an unknown nuclear factor. The 101 bp MEE sequence identified by promoter deletion analysis and the three fragments tested by EMSA (MEE1 to MEE3) are schematically depicted on top. The EMSA was carried out using radiolabelled MEE2 and parasite nuclear extract (results for MEE1 and MEE3 EMSAs were negative and are not shown). Competition was carried out in presence of a 5-, 25- and 100-fold molar excess of unlabelled DNA. B. Mutational analysis of MEE2. The EMSA was carried out using radiolabelled MEE2 and parasite nuclear extract. Competition was carried out in presence of a 25- and 100-fold molar excess of unlabelled DNA. The nucleotide sequences of wild-type and mutated MEE2 elements are indicated on the right. The ATAGATTA core motif is underlined. Mutated 8mers are highlighted in red (see Fig. S3A for competition with MEE2-mut6). The MEE2-related element upstream of PF07_0048 is shown at the bottom and differences compared with MEE2 are highlighted in red.

The proposed function of the MEE in mutually exclusive activation may be directly linked to the specific recruitment of an unknown regulatory factor. We therefore tested three overlapping fragments (MEE1–MEE3) in electromobility shift assays (EMSA) using parasite nuclear extracts. Whereas MEE1 and MEE3 showed no sign of specific binding (data not shown), the central 47 bp MEE2 fragment formed a DNA–protein complex that was specifically competed in a dose-dependent manner by an excess of homologous competitor only (Fig. 5A). To characterize this interaction in more detail we performed competition EMSAs using a set of mutated MEE2 sequences (Fig. 5B). As expected, scrambled MEE2 failed to compete underscoring the sequence-specificity of this interaction. Four out of six fragments carrying consecutively mutated 8mers (MEE2-mut2/-mut3/-mut5/-mut6) competed with similar efficiency as the MEE2 wild-type sequence (Figs 5B and S3A). In contrast, MEE2-mut4 failed to compete even at a 100-fold molar excess, and MEE2-mut1 competed with intermediate efficiency. Hence, we conclude that the 8 bp ATAGATTA sequence mutated in MEE2-mut4 represents a core motif necessary for this specific interaction, whereas the 8mer sequence at the 5′ end of MEE2 may have ancillary function in complex formation.

Next, we asked if the MEE2 element also occurs upstream of other *var* genes. We inspected all *var* upstream sequences (−600 to −1 relative to the start ATG) and identified a perfect or slightly deviated MEE2 core motif with the consensus sequence (A/T)(A/T)(A/T)GA(T/A)TA in 44 (73%) out of all 60 *var* genes. Strikingly, in all but four cases this motif (i) is conserved in terms of orientation and position relative to the ATG start codon, (ii) is embedded in an overall highly similar sequence context including a characteristic poly-dT stretch, and (iii) occurs in upsB-, upsC-, upsB/C- and upsB/A-type *var* genes (Fig. S4). The remaining four core motifs were found in one upsB/C and three upsA-type upstream sequences but they did not share these characteristics; they occurred in a different sequence context and relative position/orientation. In EMSA experiments, the MEE2-like motif derived from another upsC *var* gene (PF07_0048), in which six nucleotide positions are changed compared with MEE2 including one substitution in the core motif, competed as efficiently as the wild-type MEE2 motif (Fig. 5B). Similarly, the element found upstream of an upsB-type *var* gene (PFL0005w), in which 19 positions are altered including two in the core motif, competed albeit with lower efficiency (Fig. S3B). In contrast, competitors derived from a *var* upsA (PFD1235w) and a *var*-unrelated *rif* (PFB0035c) upstream region, in which an AT(A/T)GATTA core motif is present at the same relative position as in MEE2, failed to inhibit formation of the MEE2–protein complex (Fig. S3C).

Together, our results show that the MEE2-interacting factor (MIF) also binds to related motifs found in a large proportion of *var* upstream regions. Interestingly, however, MIF does not bind to unrelated sequences that contain a perfectly conserved 8 bp MEE2 core motif. Hence, this core motif is necessary but not sufficient for binding and the local *var* upstream sequence context plays an important role in mediating stable and sequence- specific complex formation.

## Discussion

The importance of mutually exclusive transcription of gene families is exemplified by antigenic variation in unicellular pathogens as a prime strategy to secure survival and transmission. In *T. brucei*, the causing agent of African sleeping sickness, mutually exclusive transcription of variant surface glycoprotein genes is carried out by an extranucleolar RNA polI-containing body (Navarro and Gull, 2001). Another paradigm of mutual exclusion is that of singular odorant receptor (OR) gene choice in individual olfactory neurones in mammals (McClintock, 2010). Here, exclusive transcription of one out of over a thousand OR genes involves regulatory DNA elements both upstream and in the coding regions (Qasba and Reed, 1998; Vassalli *et al*., 2002; Lomvardas *et al*., 2006; Fuss *et al*., 2007; Nguyen *et al*., 2007), and a negative protein feedback mechanism (Serizawa *et al*., 2003; Lewcock and Reed, 2004; Shykind *et al*., 2004). In addition, and in remarkable analogy to mutually exclusive *var* regulation, Lomvardas and colleagues recently described a functional association of H3K9me3 and H3K4me3 with silenced and active OR loci respectively (Magklara *et al*., 2011). These important discoveries notwithstanding, we still lack detailed knowledge as to how mutually exclusive transcription is achieved in any system. In this study, we developed and successfully applied a complementary functional approach to study mutual exclusion in *P. falciparum var* gene transcription. For the first time, we identified *cis*-acting entities as important mediators of *var* gene activation and singular gene choice.

*var* gene transcription is mediated by RNA polII and occurs stage-specifically by activation in ring-stage parasites and subsequent repression or poising during the rest of the IDC (Kyes *et al*., 2007; Lopez-Rubio *et al*., 2007). Here, we identified a UAS element essential for upsC promoter activation. The position of this element upstream of the natural TSS, and the competence to activate transcription from a heterologous promoter, are attributes inherently associated with the role of UAS elements in transcriptional activation (Levine and Tjian, 2003). Our results are therefore consistent with the sequence-specific recruitment of a transcriptional activator by the UAS to orchestrate the assembly of the pre-initiation complex (PIC) and/or to activate RNA polII-dependent transcription. Interestingly, the fact that this element functions autonomously in a euchromatic context implies a ubiquitous rather than spatially restricted distribution of the transcriptional activator involved, which somewhat precludes a restricted role for this factor in mutually exclusive *var* activation.

The current model of mutually exclusive *var* transcription postulates the existence of a physically restricted perinuclear zone dedicated to the expression of a single *var* gene (Duraisingh *et al*., 2005; Ralph *et al*., 2005; Voss *et al*., 2006; 2007; Dzikowski *et al*., 2007; Lopez-Rubio *et al*., 2009). Activation requires entry into this zone with concomitant substitution of the formerly active locus, linked to the removal of H3K9me3/PfHP1 and deposition of H3K9ac and H3K4me2/3 marks predominantly along the region downstream of the TSS (Lopez-Rubio *et al*., 2007; Perez-Toledo *et al*., 2009). We identified a deletion downstream of the TSS as the common denominator of all four promoter variants that escaped mutually exclusive activation. Unlike full-length promoters, activation of promoters lacking this region did not occur at the expense of, but in parallel to, the transcription of an endogenous *var* gene. Notably, this deletion did not alter the relative activity of the promoter showing that the processes of promoter activation and mutually exclusive recognition are uncoupled from each other. The specific binding of a nuclear factor or complex (MIF) to a *cis*-acting sequence motif present in this region (MEE2) corroborates this hypothesis and suggests an important role for this DNA–protein interaction in mutually exclusive promoter activation. The presence of MEE2-related motifs in a large subset of *var* genes provides circumstantial evidence for a conserved mechanism of singular *var* gene choice. Although the exact function of this interaction remains to be discovered, binding of MIF to the mutual exclusion element may earmark *var* loci for mutually exclusive activation. Additional experiments tailored towards identifying MIF and dissecting the exact function of this interaction in *var* regulation are now required to test this hypothesis. In this context it is worth mentioning that the 47 bp MEE2 sequence does not contain any obvious ApiAP2 transcription factor-binding motifs (Campbell *et al*., 2010).

**Fig. 6:**
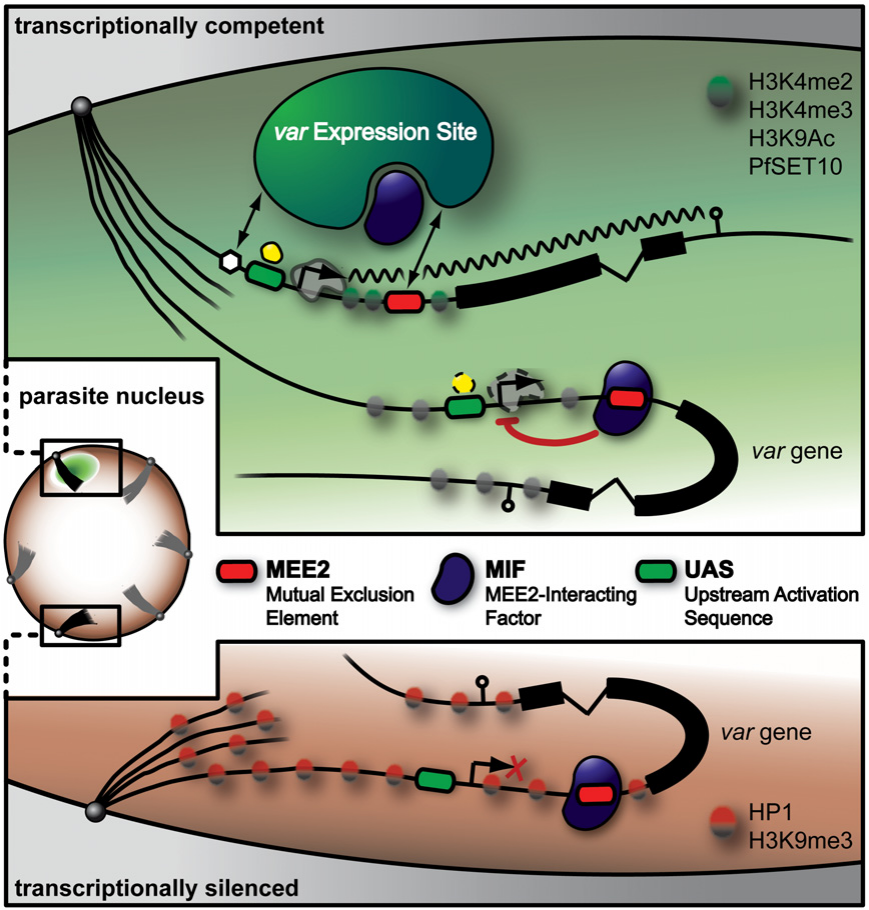
A novel model for singular *var* gene choice. A chromosome end cluster located in a transcriptionally permissive perinuclear region is schematically depicted on top. The unique *var* gene expression site (VES) recognizes a single *var* gene through specific interaction with unknown DNA motifs (white hexagon) and/or the MEE2 element itself (red oval). This interaction leads to dissociation of the MIF complex (blue) concomitant with the establishment of a permissive chromatin conformation (green circles) to facilitate RNA polII-dependent transcriptional initiation and/or elongation. This process involves deposition and maintenance of permissive histone modifications through modifying enzymes such as PfSET10 (Volz *et al*., 2012) as well as interactions between unknown transcription factors (yellow) and the UAS (green oval). Additional *var* genes within this subnuclear domain are excluded from the VES and protected from illegitimate transcription. Here, the function of MIF may be to block transcriptional elongation or to prevent transcriptional initiation or PIC assembly on the core promoter. *var* genes in heterochromatic perinculear zones that are silenced primarily through their association with H3K9me3/PfHP1 are shown below.

Using promoter deletion analyses combined with ectopic insertion of *var* elements into a euchromatic locus we were able to systematically reconstruct some of the control steps of *var* gene activation and mutual exclusion. Based on these novel findings, and by integrating current knowledge, we propose a speculative mechanistic model for mutually exclusive *var* gene activation (Fig. 6). The position of *var* loci in heterochromatic perinuclear clusters prevents accessibility to specific and general transcription factors and this is probably the most important determinant of transcriptional inactivity (Freitas-Junior *et al*., 2000; 2005; Duraisingh *et al*., 2005; Ralph *et al*., 2005; Voss *et al*., 2006; Flueck *et al*., 2009; Lopez-Rubio *et al*., 2009; Perez-Toledo *et al*., 2009). The MEE2-interacting factor or complex MIF may bind downstream of the TSS to reinforce repression and/or to prevent or reduce leaky transcription from silenced loci. Such a function may be crucial in keeping *var* genes repressed that are positioned within euchromatic zones at the nuclear periphery (Ralph *et al*., 2005). Singular *var* gene choice may occur through the recognition of the MEE2/MIF complex, or an alternative *var* locus-specific sequence tag, by the unique *var* gene expression site (VES) (Duraisingh *et al*., 2005; Voss *et al*., 2006; Dzikowski *et al*., 2007; Lopez-Rubio *et al*., 2009). Once locked in, the VES may trigger the exchange of H3K9me3/PfHP1 with H3K4me2/3 and H3K9ac marks and the dissociation of the repressive MIF complex. Physical association of the active *var* locus with the VES may also play a crucial role in epigenetic memory, i.e. in keeping the *var* gene in place for re-activation in daughter cells (Lopez-Rubio *et al*., 2007). In this context, it is tempting to speculate that the recently identified histone methyltransferase PfSET10 (Volz *et al*., 2012) may be one component of the VES compartment.

This model proposes a novel logic in mutually exclusive gene expression and provides us with an informed working hypothesis for further functional dissection of the mechanisms orchestrating singular *var* gene choice. In particular, targeted identification of the proteins or protein complexes interacting with the regulatory elements characterized in this study will be a promising and exciting avenue to pursue. Detailed insight into this complex regulatory system is important for our understanding of immune evasion and virulence of *P. falciparum* and other pathogens. Furthermore, our results will also help to understand conceptually similar processes in other organisms.

## Experimental procedures

### Parasite culture and transfection

*Plasmodium falciparum* 3D7 parasites were cultured as described previously (Trager and Jenson, 1978). Growth synchronization was achieved by repeated sorbitol lysis (Lambros and Vanderberg, 1979). Transfections were performed as described (Voss *et al*., 2006). Parasites were selected on 2.5 μg ml^−1^ blasticidin-S-HCl and 4 nM WR99210. Transfection constructs are described in supporting experimental procedures.

### Quantitative reverse transcription PCR

qPCR was performed on reverse transcribed total RNA and gDNA isolated from synchronous parasite cultures. A detailed protocol, relative transcript calculation and primer sequences are provided in supporting experimental procedures and Table S1.

### Southern and Northern blot analysis

gDNA was digested with appropriate restriction enzymes overnight and separated in 0.5× TBE-buffered 0.7% agarose gels. Total RNA was isolated from saponin-released parasites using TriReagent (Ambion). RNA was glyoxylated for 1 h at 55°C in five volumes glyoxal reaction mixture and electrophoresis was performed using 1× BPTE-buffered 1.5% agarose gels (Sambrook and Russell, 2001). Blots were probed with ^32^P-dATP-labelled h*dhfr*, *kahrp* and *hsp86* PCR fragments. Membranes were stripped by boiling in 0.1% SDS for 15 min in between hybridizations.

### Western blot analysis

Detection of hDHFR-GFP and GAPDH (loading control) was performed on whole-cell lysates. Primary antibody dilutions were: mouse anti-GFP (Roche Diagnostics, 11814460001), 1:500; mouse anti-GAPDH 1-10B (kind gift of Claudia Daubenberger), 1:20 000. PfEMP1 was extracted from trophozoite-infected RBC pellets (Triton X-100-insoluble/SDS soluble fraction) as described (van Schravendijk *et al*., 1993). Extracts were separated by SDS-PAGE using 5% polyacrylamide gels using Tris-glycine or Tris-acetate buffers. PfEMP1 was detected using the monoclonal mouse anti-PfEMP1 antibody 1B/6H-1 (Duffy *et al*., 2002), 1:500.

### Electromobility shift assay

High-salt nuclear extracts and EMSAs were prepared and carried out as described (Voss *et al*., 2002) with the following modifications. Proteins were extracted with 500 mM KCl and incubated with 20 fmol of radiolabelled probe in 1× EMSA buffer in presence of 200 ng of poly(dA-dT) as non-specific competitor. Complementary oligonucleotide sequences used to generate double-stranded probes and competitors are listed in Table S1.

## Supporting information

**Supporting experimental procedures**

**Fig. S1.** Southern analysis of gDNA isolated from parasites presented in Fig. 2.

A. Autoradiographs of Southern blots showing episomal maintenance or plasmid integration into the endogenous *kahrp* locus in 3D7/pBK_min_ and 3D7/pBK_min_-RI. gDNA was digested with BglII and HindIII. Blots were probed with a radiolabelled *kahrp* fragment. E, episomal; I, integrated.

B. Autoradiographs of Southern blots showing episomal maintenance or plasmid integration into the endogenous *kahrp* locus in 3D7/pBC1K_min_, 3D7/pBC2K_min_ and 3D7/pBC3K_min_. gDNA was digested with BglII and HindIII. Blots were probed with a radiolabelled *kahrp* fragment.

C. Schematic map of the endogenous *kahrp* locus.

D–F. Schematic maps of the integration events in 3D7/pBK_min_ (D), 3D7/pBC1K_min_ and 3D7/pBC2K_min_ (E) and 3D7/pBC3K_min_ (F). BglII and HindIII restriction sites and length of the corresponding fragments are indicated.

**Fig. S2.** Transcriptional initiation form an alternative upsC upstream TSS. The promoters in pBC and pBC4 are schematically depicted on top. Semi-quantitative analysis of protein and transcript abundance by Western and Northern blot in a time-course experiment. Total protein and RNA were harvested simultaneously from synchronized 3D7/pBC and 3D7/pBC4 parasites at three consecutive time points during intra-erythrocytic development (ring stages, 8–18 hpi; late ring stages/early trophozoites, 16–26 hpi; late trophozoites/early schizonts, 24–34 hpi). Expression of hDHFR-GFP and GAPDH (loading control) was detected with anti-GFP and anti-GAPDH antibodies respectively (upper panels). Steady-state h*dhfr-gfp* and *hsp86* (loading control) transcripts were detected using radiolabelled h*dhfr* and *hsp86* probes respectively.

**Fig. S3.** Competition EMSAs. All EMSAs were carried out using radiolabelled MEE2 and parasite nuclear extract.

A. Mutational analysis of MEE2. Competition was carried out in presence of a 25- and 100-fold molar excess of unlabelled DNA. The nucleotide sequences of wild-type and mutated MEE2 elements are indicated on the right. The ATAGATTA core motif is underlined. Mutated 8mers are highlighted in red.

B. Competition of the MEE2 complex by a MEE2-related upsB sequence element. Competition was carried out in presence of a 25-, 100-, 250- and 500-fold molar excess of unlabelled DNA. The nucleotide sequences of wild-type and scrambled MEE2 and the MEE2-related upsB element are indicated on the right. The ATAGATTA core motif is underlined. The differences in the upsB-derived motif compared with MEE2 are highlighted in red.

C. The ATAGATTA core motif is not sufficient for complex formation. Competition was carried out in presence of a 25-, 100- and 500-fold molar excess of unlabelled DNA. The ATAGATTA core motif is underlined. The nucleotide sequences of wild-type and scrambled MEE2 and two unrelated sequence elements that contain the ATAGATTA core motif are indicated on the right.

**Fig. S4.** The MEE2 core motif occurs in a conserved position upstream of 44 *var* genes.

A. The schematic shows the presence and relative position of the (A/T)(A/T)(A/T)GA(A/T)TA consensus sequence found upstream of 44 *var* genes. This motif forms the core of the 47 bp MEE2 element that is bound by a nuclear factor in a sequence-specific manner (see Figs 4 and S3). Red boxes indicate the position of the motif in each upstream region. Numbers on the right represent the position of the first nucleotide of the motif relative to the translation initiation ATG. Gene accession numbers were retrieved from PlasmoDB version 7.2 (http://www.plasmoDB.org) and are indicated on the left. The colour code clusters *var* genes into the different *var* gene subgroups upsA, upsB, upsC, upsE, upsB/C and upsB/A (Lavstsen *et al*., 2003).

B. Alignment of MEE2-related sequences that are centred around the (A/T)(A/T)(A/T)GA(A/T)TA core consensus element in 44 *var* upstream regions. The original MEE2 motif identified upstream of the upsC *var* gene PFL1960w is shown as the first sequence in the alignment. The local context of the MEE2-related core motifs shows a high level of sequence similarity that includes a prominent upstream poly-dT stretch. Gene accession numbers are indicated on the left and are colour-coded as in Fig. S4A. Orientation of the motif is indicated on the right (+, upper strand; −, lower strand). The red bar on top highlights the position of the core motif.

**Table S1.** All primers used in this study are listed. Restriction sites are indicated in bold.
