# Supplementary figures and images for: Identification of a cis-acting DNA–protein interaction implicated in singular var gene choice in Plasmodium falciparum
Source: Cell Microbiol. 2012 Sep 4;14(12):1836–48. doi: 10.1111/cmi.12004 (PMC3549481; doi:10.1111/cmi.12004)

Figure S1\_Brancucci et al.

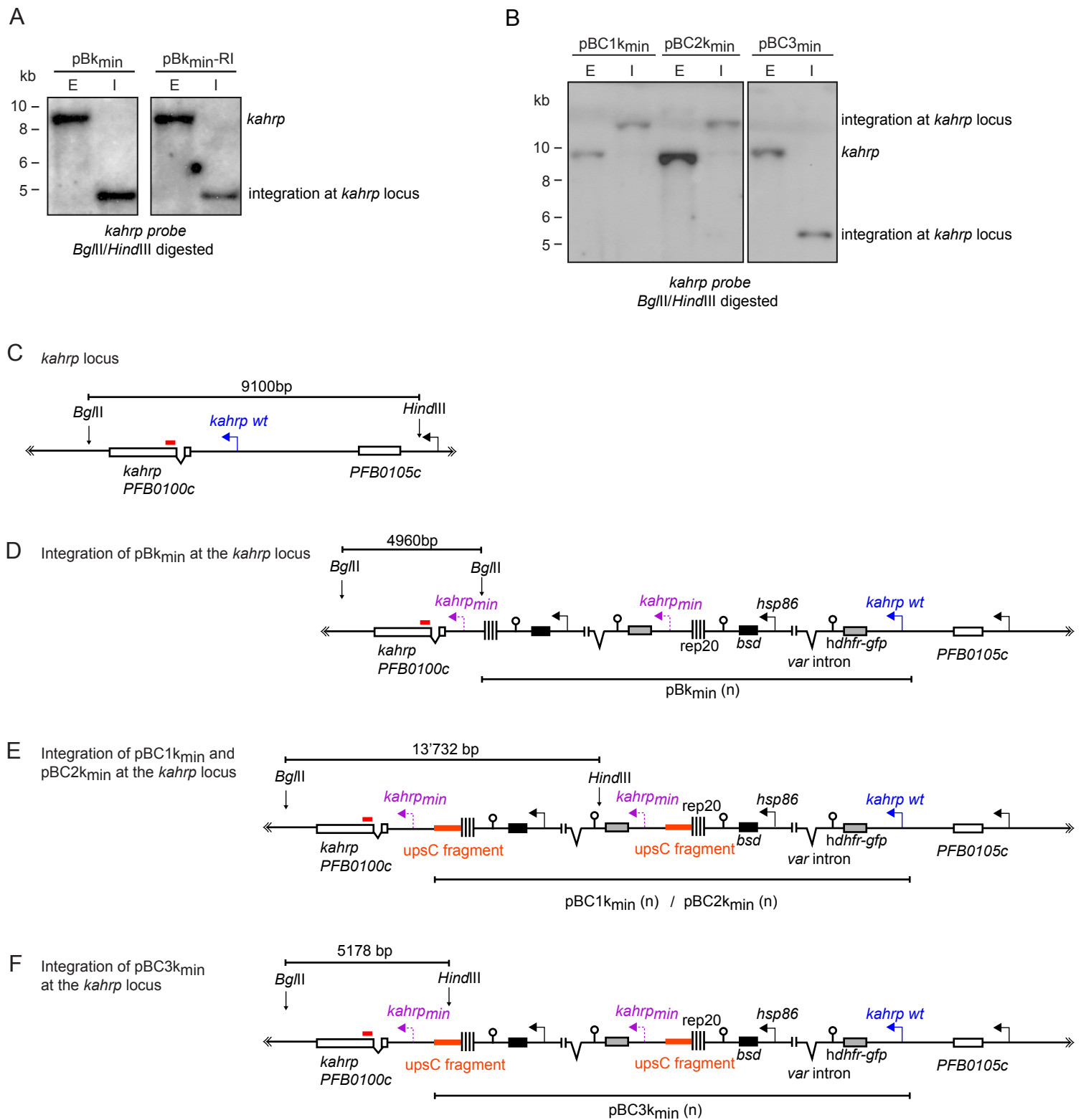

Supplement: Supplementary file 1 [file cmi0014-1836-SD1.pdf]

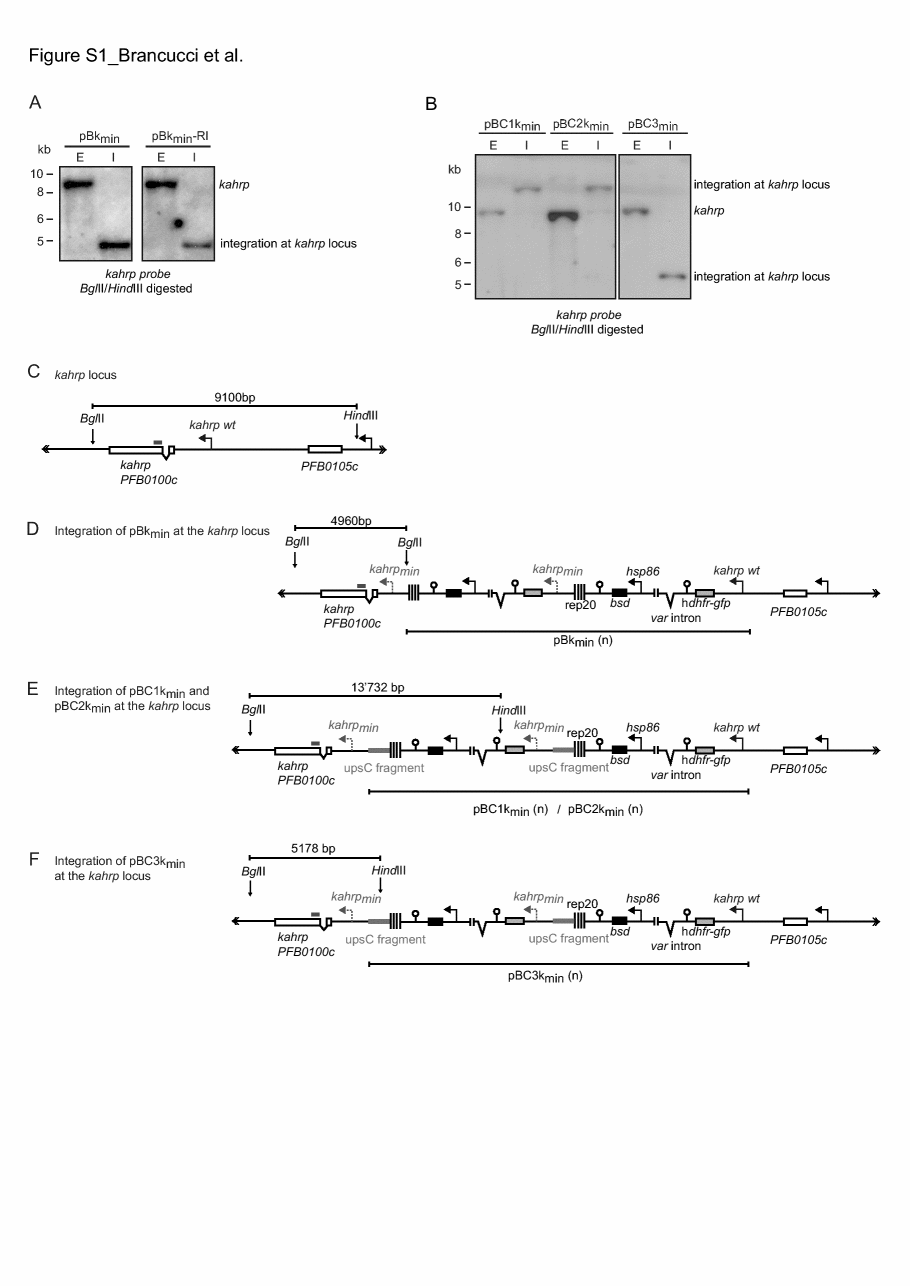

Supplement: Supplementary file 2 [file cmi0014-1836-SD6.png]

Figure S2\_Brancucci et al.

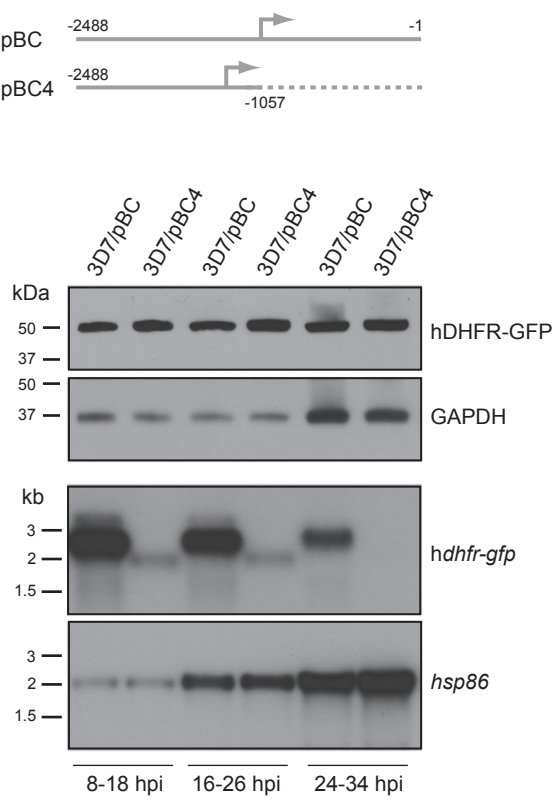

Supplement: Supplementary file 5 [file cmi0014-1836-SD2.pdf]

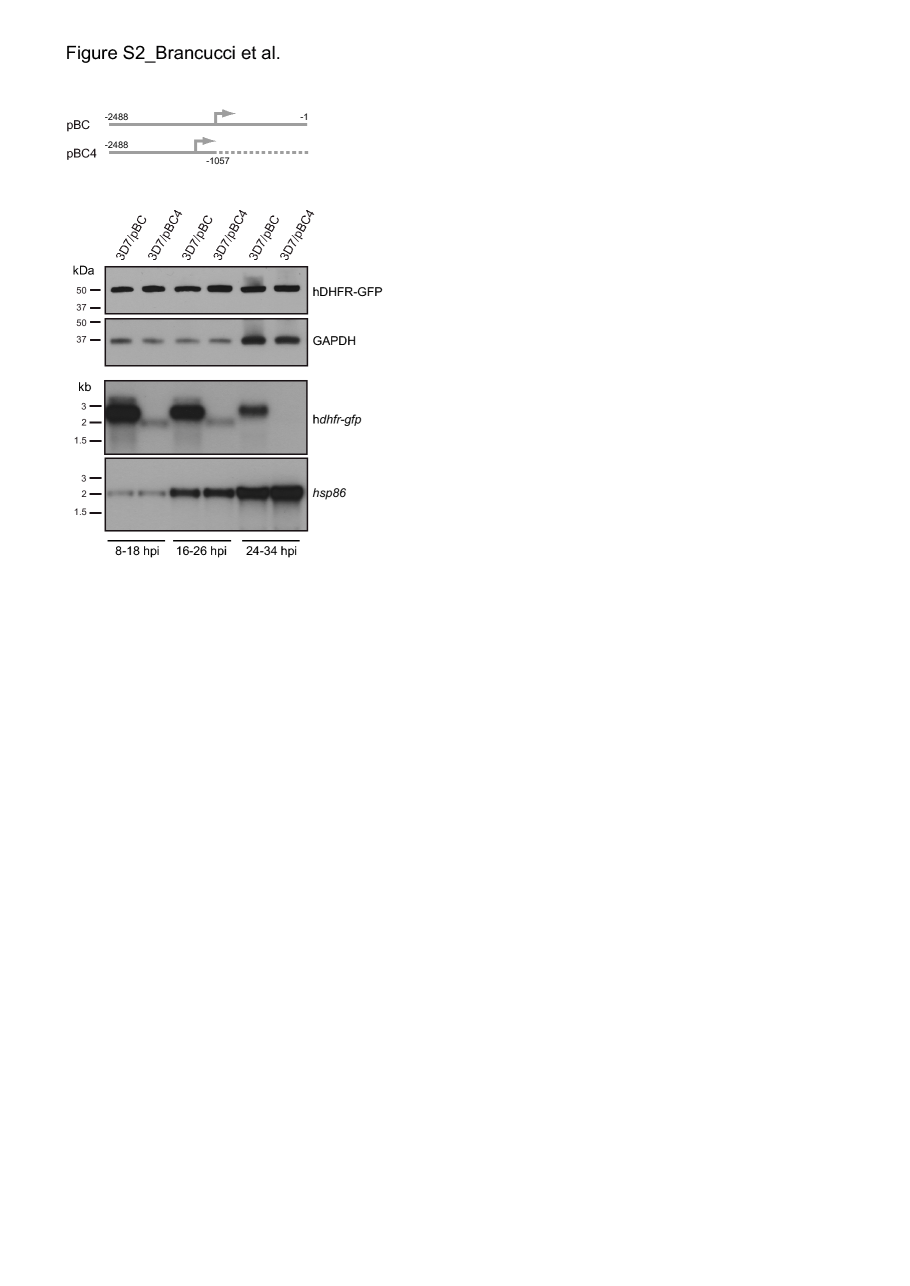

Supplement: Supplementary file 6 [file cmi0014-1836-SD7.png]

Figure S3\_Brancucci et al.

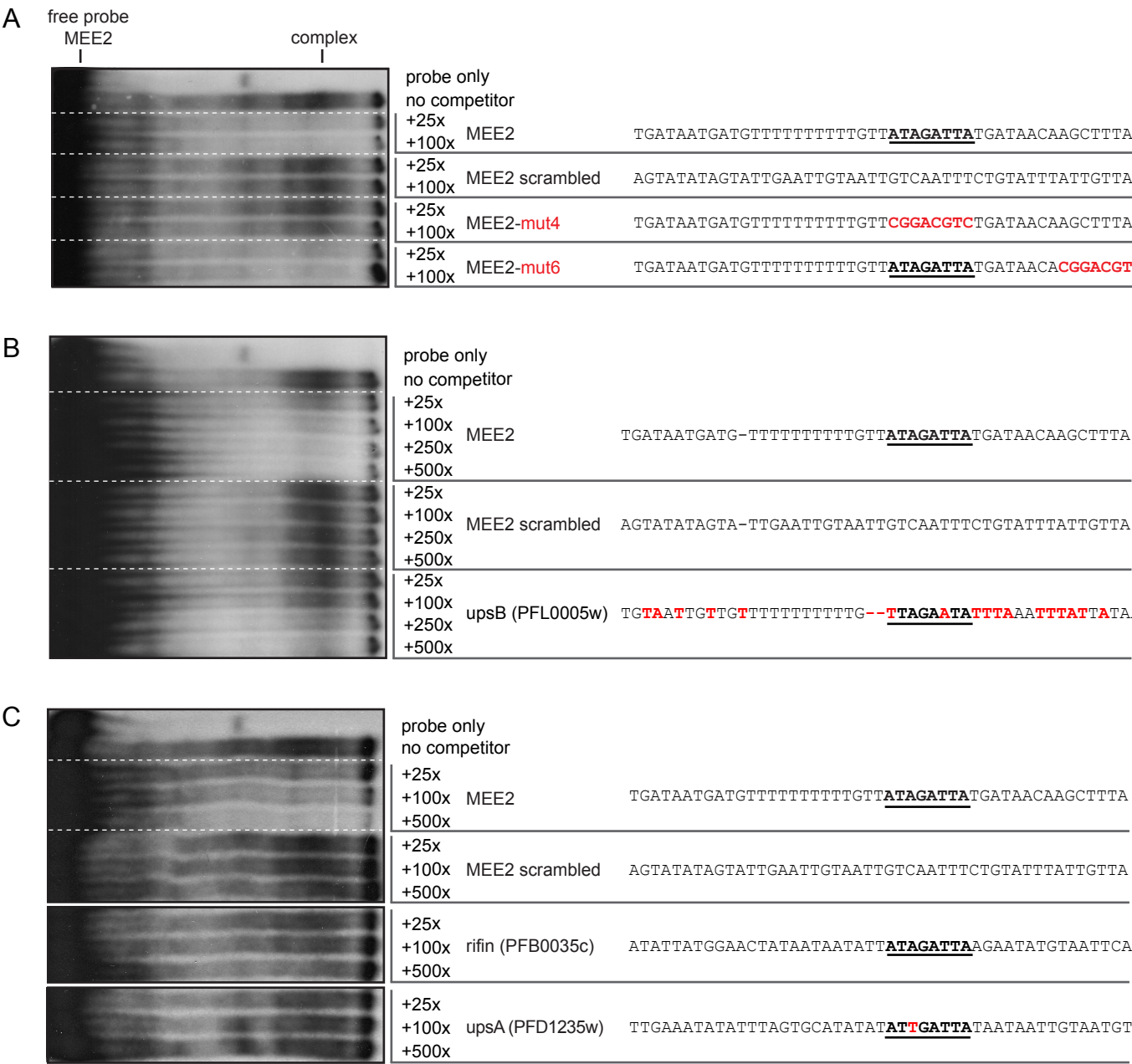

Supplement: Supplementary file 8 [file cmi0014-1836-SD3.pdf]

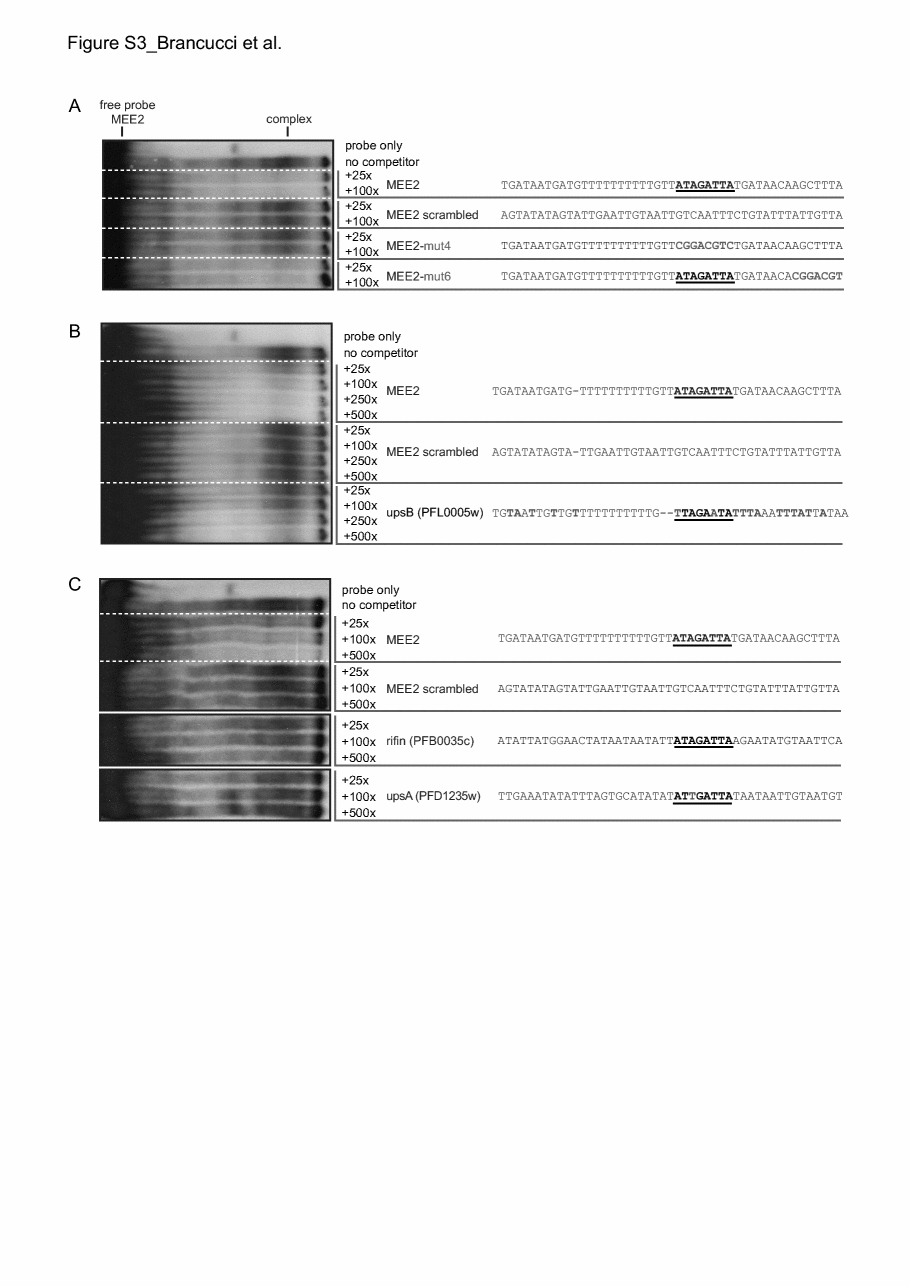

Supplement: Supplementary file 9 [file cmi0014-1836-SD8.png]

A

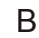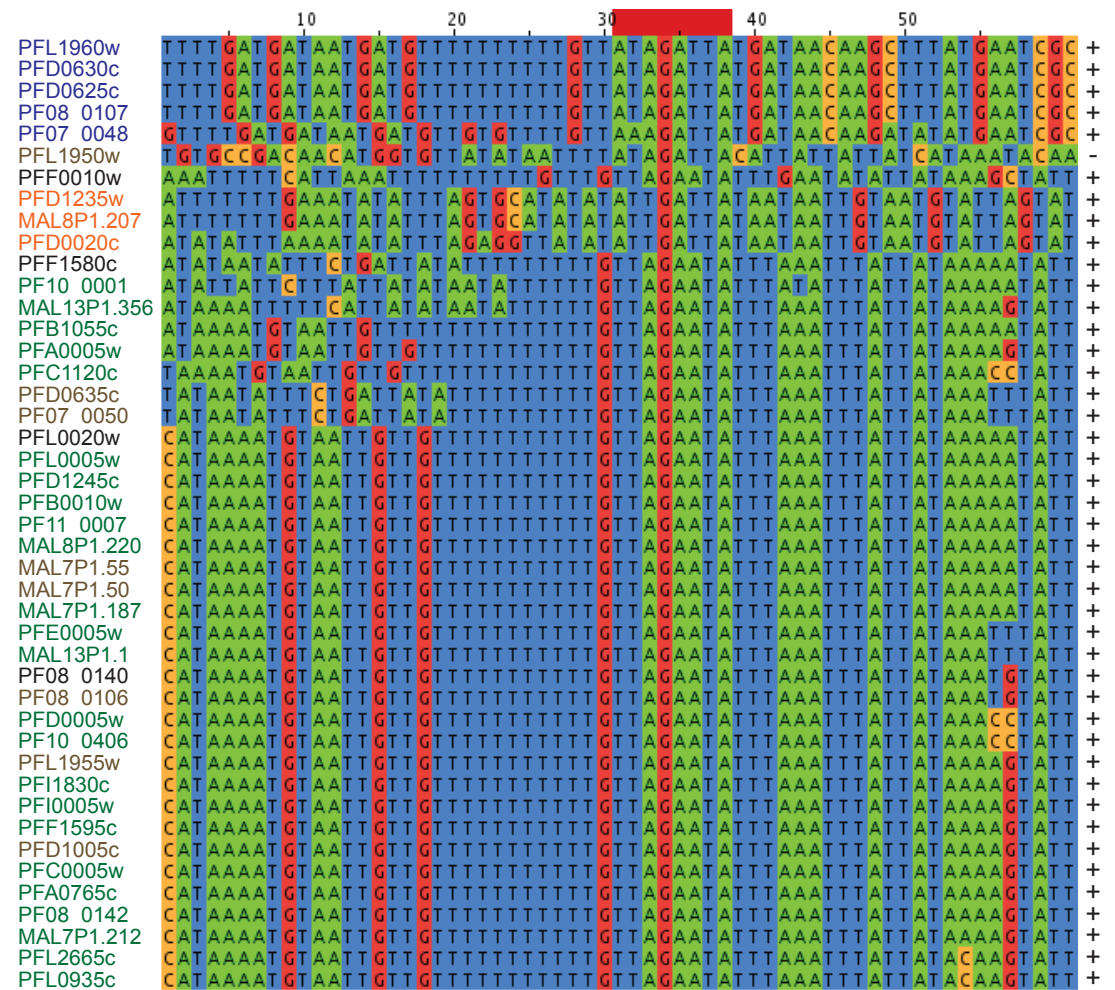

Supplement: Supplementary file 11 [file cmi0014-1836-SD4.pdf]

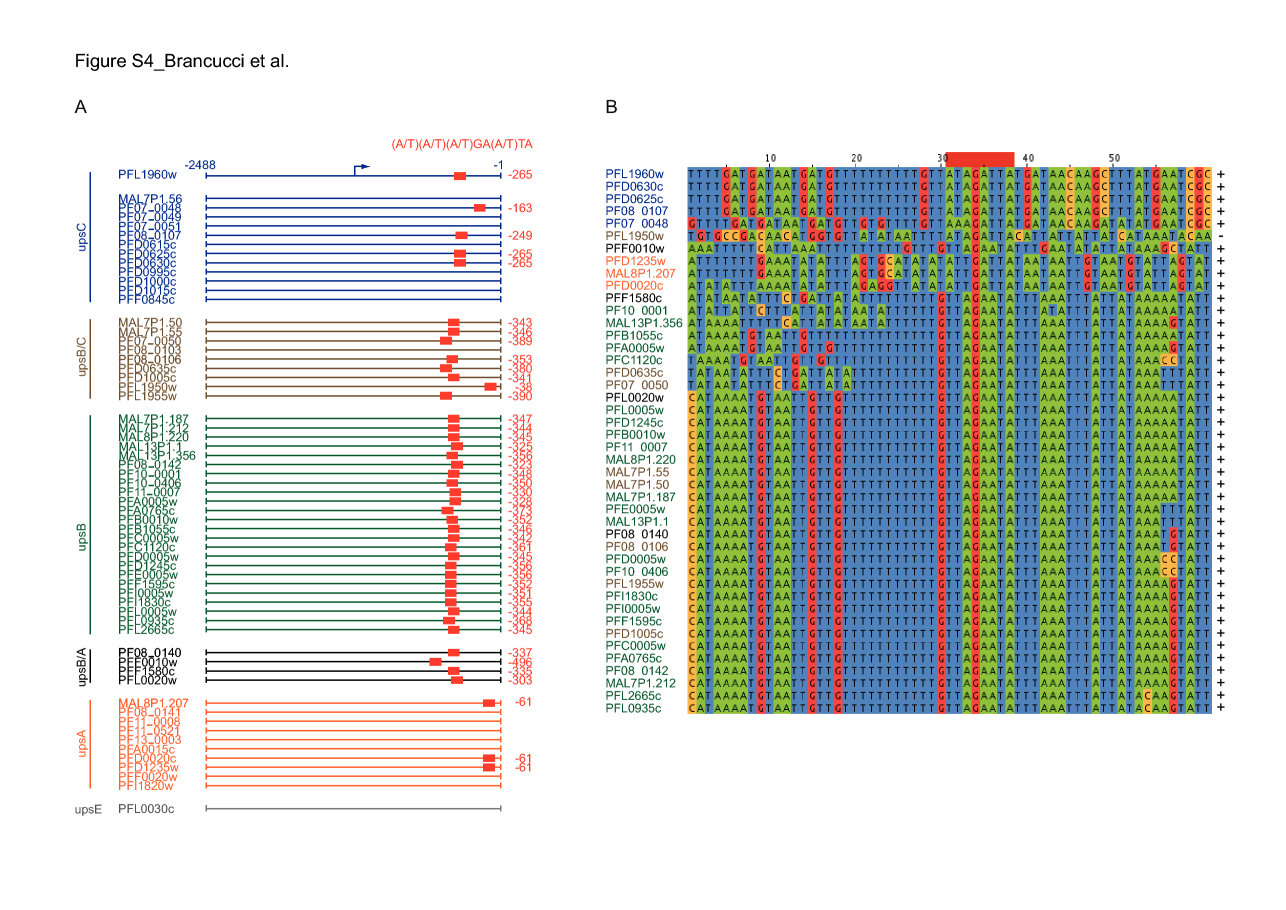

Supplement: Supplementary file 12 [file cmi0014-1836-SD9.png]

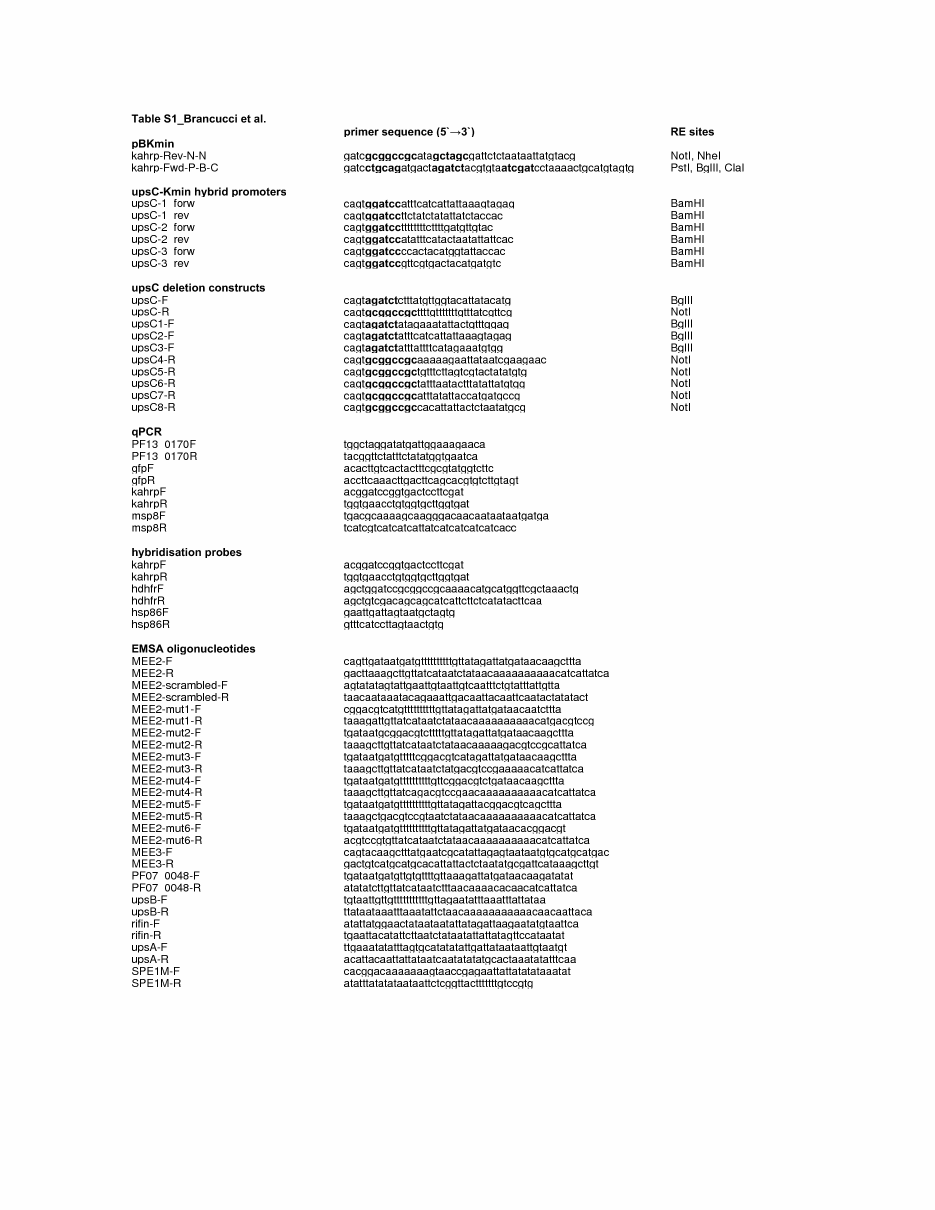

Supplement: Supplementary file 15 [file cmi0014-1836-SD10.png]
