# Supplementary material for: Identification of a cis-acting DNA–protein interaction implicated in singular var gene choice in Plasmodium falciparum
Source: Cell Microbiol. 2012 Sep 4;14(12):1836–48. doi: 10.1111/cmi.12004 (PMC3549481; doi:10.1111/cmi.12004)
Supplement: Supplementary file 4 [file cmi0014-1836-SD16.doc]

**Supporting Information for:**

**Identification of a *cis*-acting DNA-protein interaction implicated in singular *var* gene choice in *Plasmodium falciparum***

Nicolas M. B. Brancucci, Kathrin Witmer, Christoph D. Schmid, Christian Flueck, and Till S. Voss

**Supporting Experimental Procedures**

**Transfection constructs**

All transfection constructs generated in this study are derivatives of pBK_min_ that was itself obtained by replacing the *cam* promoter in pBcam (Witmer et al., 2012) with the 1115bp minimal *kahrp* promoter (K_min_) amplified from 3D7 gDNA and *Bgl*II/*Not*I restriction. Constructs pBC and pBC1 to pBC8 were generated by replacing K_min_ with *Bgl*II/*Not*I-digested upsC upstream sequences (PFL1960w) amplified from pCAT5B1 (Voss et al., 2000). upsC-*kahrp* hybrid promoters were obtained by cloning *BamH*I-digested upsC fragments into *Bgl*II-digested pBK_min_. The *mahrp1* promoter was amplified from gDNA and cloned into *Bgl*II/*Not*I-digested pBK_min_ to obtain pBM. Importantly, the parental vector pBcam contains the 5bp AAAACA sequence, which naturally occurs directly upstream of the PFL1960w *var* ATG, upstream of the h*dhfr-gfp* ATG. This ensures an identical sequence context for translational initiation for all constructs. All primer sequences are listed in Table S1.

**Quantitative reverse transcription PCR**

Pre-synchronised parasite cultures were synchronised twice 16 hours apart to obtain an eight hour growth window. Total RNA was isolated using Tri Reagent (Ambion) and further purified using the RNeasy Plus Mini Kit (Qiagen) for removal of gDNA. Residual gDNA was digested with TURBO DNA-*free*^TM^ (Ambion). All samples were tested negative for contaminating gDNA by qPCR. RNA was reverse transcribed using the RETROscript Kit (Ambion). qPCR reactions for absolute transcript quantification of h*dhfr-gfp*, *kahrp,* PF13_0170 (glutaminyl-tRNA synthetase) and *msp8* were performed at final primer concentrations of 0.4μM using SYBR Green Master Mix (Applied Biosystems) on a StepOnePlus^TM^ Real-Time PCR System (Applied Biosystems) in a reaction volume of 12ul. Plasmid copy numbers were determined by qPCR on gDNA isolated from the same parasite samples and calculated by dividing the absolute h*dhfr-gfp* copy numbers by the average value obtained for *msp8* and PF13_0170. All reactions were run in duplicate or triplicate yielding virtually identical Ct values. Serial dilutions of gDNA and plasmid DNA were used as standards for absolute quantification. Relative “transcripts per parasite” were calculated by normalisation against the house-keeping gene PF13_0170 or *msp8*. Relative “transcripts per promoter” were calculated by dividing the relative “transcripts per parasite” by the average number of plasmid copies. Primer sequences are listed in Table S1.

**Supporting Figure and Table legends**

**Fig. S1.** Southern analysis of gDNA isolated from parasites presented in Fig. 2.

A. Autoradiographs of Southern blots showing episomal maintenance or plasmid integration into the endogenous *kahrp* locus in 3D7/pBK_min_ and 3D7/pBK_min_-RI. gDNA was digested with *Bgl*II and *Hind*III. Blots were probed with a radiolabeled *kahrp* fragment. E, episomal; I, integrated.

B. Autoradiographs of Southern blots showing episomal maintenance or plasmid integration into the endogenous *kahrp* locus in 3D7/pBC1K_min_, 3D7/pBC2K_min_ and 3D7/pBC3K_min_. gDNA was digested with *Bgl*II and *Hind*III. Blots were probed with a radiolabeled *kahrp* fragment.

C. Schematic map of the endogenous *kahrp* locus.

D-F. Schematic maps of the integration events in 3D7/pBK_min_ (D), 3D7/pBC1K_min_ and 3D7/pBC2K_min_ (E), and 3D7/pBC3K_min_ (F). *Bgl*II and *Hind*III restriction sites and length of the corresponding fragments are indicated.

**Fig. S2.** Transcriptional initiation form an alternative upsC upstream TSS.

The promoters in pBC and pBC4 are schematically depicted on top. Semi-quantitative analysis of protein and transcript abundance by Western and Northern blot in a time-course experiment. Total protein and RNA were harvested simultaneously from synchronised 3D7/pBC and 3D7/pBC4 parasites at three consecutive time points during intra-erythrocytic development (ring stages, 8-18hpi; late ring stages/early trophozoites, 16-26hpi; late trophozoites/early schizonts, 24-34hpi). Expression of hDHFR-GFP and GAPDH (loading control) was detected with anti-GFP and anti-GAPDH antibodies, respectively (upper panels). Steady-state h*dhfr-gfp* and *hsp86* (loading control) transcripts were detected using radiolabeled h*dhfr* and *hsp86* probes, respectively.

**Fig. S3.** Competition EMSAs. All EMSAs were carried out using radiolabeled MEE2 and parasite nuclear extract.

A. Mutational analysis of MEE2. Competition was carried out in presence of a 25- and 100-fold molar excess of unlabeled DNA. The nucleotide sequences of wild-type and mutated MEE2 elements are indicated on the right. The ATAGATTA core motif is underlined. Mutated 8mers are highlighted in red.

B. Competition of the MEE2 complex by a MEE2-related upsB sequence element. Competition was carried out in presence of a 25-, 100-, 250- and 500-fold molar excess of unlabeled DNA. The nucleotide sequences of wild-type and scrambled MEE2 and the MEE2-related upsB element are indicated on the right. The ATAGATTA core motif is underlined. The differences in the upsB-derived motif compared to MEE2 are highlighted in red.

C. The ATAGATTA core motif is not sufficient for complex formation. Competition was carried out in presence of a 25-, 100- and 500-fold molar excess of unlabeled DNA. The ATAGATTA core motif is underlined. The nucleotide sequences of wild-type and scrambled MEE2 and two unrelated sequence elements that contain the ATAGATTA core motif are indicated on the right.

**Fig. S4.** The MEE2 core motif occurs in a conserved position upstream of 44 *var* genes.

A. The schematic shows the presence and relative position of the (A/T)(A/T)(A/T)GA(A/T)TA consensus sequence found upstream of 44 *var* genes. This motif forms the core of the 47bp MEE2 element that is bound by a nuclear factor in a sequence-specific manner (see Figs. 4 and S3). Red boxes indicate the position of the motif in each upstream region. Numbers on the right represent the position of the first nucleotide of the motif relative to the translation initiation ATG. Gene accession numbers were retrieved from PlasmoDB version 7.2 ([www.plasmoDB.org](http://www.plasmoDB.org/)) and are indicated on the left. The colour code clusters *var* genes into the different *var* gene subgroups upsA, upsB, upsC, upsE, upsB/C, and upsB/A (Lavstsen et al., 2003).

B. Alignment of MEE2-related sequences that are centred around the (A/T)(A/T)(A/T)GA(A/T)TA core consensus element in 44 *var* upstream regions. The original MEE2 motif identified upstream of the upsC *var* gene PFL1960w is shown as the first sequence in the alignment. The local context of the MEE2-related core motifs shows a high level of sequence similarity that includes a prominent upstream poly-dT stretch. Gene accession numbers are indicated on the left and are colour-coded as in Fig. S4A. Orientation of the motif is indicated on the right (+, upper strand; -, lower strand). The red bar on top highlights the position of the core motif.

**Table S1.** All primers used in this study are listed. Restriction sites are indicated in bold.

**References**

Lavstsen, T., Salanti, A., Jensen, A. T., Arnot, D. E., and Theander, T. G. (2003) Sub-grouping of Plasmodium falciparum 3D7 var genes based on sequence analysis of coding and non-coding regions. *Malar J* **2:** 27.

Voss, T. S., Thompson, J. K., Waterkeyn, J., Felger, I., Weiss, N., Cowman, A. F. *et al.* (2000) Genomic distribution and functional characterisation of two distinct and conserved Plasmodium falciparum var gene 5' flanking sequences
109. *Mol Biochem Parasitol* **107:** 103-115.

Witmer, K., Schmid, C. D., Brancucci, N. M., Luah, Y. H., Preiser, P. R., Bozdech, Z. *et al.* (2012) Analysis of subtelomeric virulence gene families in Plasmodium falciparum by comparative transcriptional profiling. *Mol Microbiol* **84**: 243-59.
