# Supplementary material for: Identification of a cis-acting DNA–protein interaction implicated in singular var gene choice in Plasmodium falciparum
Source: Cell Microbiol. 2012 Sep 4;14(12):1836–48. doi: 10.1111/cmi.12004 (PMC3549481; doi:10.1111/cmi.12004)
Supplement: Supplementary file 14 [file cmi0014-1836-SD5.pdf]

Table S1_Brancucci et al.

	primer sequence (5`→3`)	RE sites
**pBKmin**		
kahrp-Rev-N-N	gatc**gcggccgc**ata**gctagc**gattctctaataattatgtacg	NotI, NheI
kahrp-Fwd-P-B-C	gatc**ctgcag**atgact**agatct**acgtgta**atcgat**cctaaaactgcatgtagtg	PstI, BglII, ClaI
**upsC-Kmin hybrid promoters**		
upsC-1 forw	cagt**ggatcc**atttcatcattattaaagtagag	BamHI
upsC-1 rev	cagt**ggatcc**ttctatctatattatctaccac	BamHI
upsC-2 forw	cagt**ggatcc**ttttttttcttttgatgttgtac	BamHI
upsC-2 rev	cagt**ggatcc**atatttcatactaatattattcac	BamHI
upsC-3 forw	cagt**ggatcc**ccactacatggtattaccac	BamHI
upsC-3 rev	cagt**ggatcc**gttcgtgactacatgatgtc	BamHI
**upsC deletion constructs**		
upsC-F	cagt**agatct**ctttatgttggtacattatacatg	BglII
upsC-R	cagt**gcggccgc**ttttgtttttttgtttatcgttcg	NotI
upsC1-F	cagt**agatct**atagaaatattactgtttggag	BglII
upsC2-F	cagt**agatct**atttcatcattattaaagtagag	BglII
upsC3-F	cagt**agatct**atttattttcatagaaatgtgg	BglII
upsC4-R	cagt**gcggccgc**aaaaagaattataatcgaagaac	NotI
upsC5-R	cagt**gcggccgc**tgtttcttagtcgtactatatgtg	NotI
upsC6-R	cagt**gcggccgc**tatttaatactttatattatgtgg	NotI
upsC7-R	cagt**gcggccgc**atttatattaccatgatgccg	NotI
upsC8-R	cagt**gcggccgc**cacattattactctaatatgcg	NotI
**qPCR**		
PF13 0170F	tggctaggatatgattggaaagaaca	
PF13 0170R	tacggttctatttctatatggtgaatca	
gfpF	acacttgtcactactttcgcgtatggtcttc	
gfpR	accttcaaacttgacttcagcacgtgtcttgtagt	
kahrpF	acggatccggtgactccttcgat	
kahrpR	tggtgaacctgtggtgcttggtgat	
msp8F	tgacgcaaaagcaagggacaacaataataatgatga	
msp8R	tcatcgtcatcatcattatcatcatcatcatcacc	
**hybridisation probes**		
kahrpF	acggatccggtgactccttcgat	
kahrpR	tggtgaacctgtggtgcttggtgat	
hdhfrF	agctggatccgcggccgcaaaacatgcatggttcgctaaactg	
hdhfrR	agctgtcgacagcagcatcattcttctcatatacttcaa	
hsp86F	gaattgattagtaatgctagtg	
hsp86R	gtttcatccttagtaactgtg	
**EMSA oligonucleotides**		
MEE2-F	cagttgataatgatgttttttttttgttatagattatgataacaagcttta	
MEE2-R	gacttaaagcttgttatcataatctataacaaaaaaaaaacatcattatca	
MEE2-scrambled-F	agtatatagtattgaattgtaattgtcaatttctgtatttattgtta	
MEE2-scrambled-R	taacaataaatacagaaattgacaattacaattcaatactatatact	
MEE2-mut1-F	cggacgtcatgttttttttttgttatagattatgataacaatcttta	
MEE2-mut1-R	taaagattgttatcataatctataacaaaaaaaaaacatgacgtccg	
MEE2-mut2-F	tgataatgcggacgtcttttgttatagattatgataacaagcttta	
MEE2-mut2-R	taaagcttgttatcataatctataacaaaaagacgtccgcattatca	
MEE2-mut3-F	tgataatgatgttttcggacgtcatagattatgataacaagcttta	
MEE2-mut3-R	taaagcttgttatcataatctatgacgtccgaaaaacatcattatca	
MEE2-mut4-F	tgataatgatgttttttttttgttcggacgtctgataacaagcttta	
MEE2-mut4-R	taaagcttgttatcagacgtccgaacaaaaaaaaaacatcattatca	
MEE2-mut5-F	tgataatgatgttttttttttgttatagattacggacgtcagcttta	
MEE2-mut5-R	taaagctgacgtccgtaatctataacaaaaaaaaaacatcattatca	
MEE2-mut6-F	tgataatgatgttttttttttgttatagattatgataacacggacgt	
MEE2-mut6-R	acgtccgtgttatcataatctataacaaaaaaaaaacatcattatca	
MEE3-F	cagtacaagctttatgaatcgcatattagagtaataatgtgcatgcatgac	
MEE3-R	gactgtcatgcatgcacattattactctaatatgcgattcataaagcttgt	
PF07 0048-F	tgataatgatgttgtgttttgttaaagattatgataacaagatatat	
PF07 0048-R	atatatcttgttatcataatctttaacaaaacacaacatcattatca	
upsB-F	tgtaattgttgttttttttttttgttagaatatttaaatttattataa	
upsB-R	ttataataaatttaaatattctaacaaaaaaaaaaaacaacaattaca	
rifin-F	atattatggaactataataatattatagattaagaatatgtaattca	
rifin-R	tgaattacatattcttaatctataatattattatagttccataatat	
upsA-F	ttgaaatatatttagtgcatatatattgattataataattgtaatgt	
upsA-R	acattacaattattataatcaatatatatgcactaaatatatttcaa	
SPE1M-F	cacggacaaaaaaagtaaccgagaattattatatataaatat	
SPE1M-R	atatttatatataataattctcggttactttttttgtccgtg	
